# Targeting Sphingosine Kinase Isoforms Effectively Reduces Growth and Survival of Neoplastic Mast Cells With D816V-KIT

**DOI:** 10.3389/fimmu.2018.00631

**Published:** 2018-03-28

**Authors:** Geethani Bandara, Rosa Muñoz-Cano, Araceli Tobío, Yuzhi Yin, Hirsh D. Komarow, Avanti Desai, Dean D. Metcalfe, Ana Olivera

**Affiliations:** ^1^Mast Cell Biology Section, Laboratory of Allergic Diseases, National Institute of Allergy and Infectious Diseases, National Institutes of Health, Bethesda, MD, United States; ^2^Allergy Section, Pneumology Department, Hospital Clinic, ARADyAL, Instituto de Salud Carlos III, Barcelona, Spain

**Keywords:** sphingosine kinase, sphingosine kinase inhibitor, sphingosine-1-phosphate, mastocytosis, D816V, KIT, mast cells

## Abstract

Mastocytosis is a disorder resulting from an abnormal mast cell (MC) accumulation in tissues that is often associated with the D816V mutation in KIT, the tyrosine kinase receptor for stem cell factor. Therapies available to treat aggressive presentations of mastocytosis are limited, thus exploration of novel pharmacological targets that reduce MC burden is desirable. Since increased generation of the lipid mediator sphingosine-1-phosphate (S1P) by sphingosine kinase (SPHK) has been linked to oncogenesis, we studied the involvement of the two SPHK isoforms (SPHK1 and SPHK2) in the regulation of neoplastic human MC growth. While SPHK2 inhibition prevented entry into the cell cycle in normal and neoplastic human MCs with minimal effect on cell survival, SPHK1 inhibition caused cell cycle arrest in G2/M and apoptosis, particularly in D816V-KIT MCs. This was mediated *via* activation of the DNA damage response (DDR) cascade, including phosphorylation of the checkpoint kinase 2 (CHK2), CHK2-mediated M-phase inducer phosphatase 3 depletion, and p53 activation. Combination treatment of SPHK inhibitors with KIT inhibitors showed greater growth inhibition of D816V-KIT MCs than either inhibitor alone. Furthermore, inhibition of SPHK isoforms reduced the number of malignant bone marrow MCs from patients with mastocytosis and the growth of D816V-KIT MCs in a xenograft mouse model. Our results reveal a role for SPHK isoforms in the regulation of growth and survival in normal and neoplastic MCs and suggest a regulatory function for SPHK1 in the DDR in MCs with KIT mutations. The findings also suggest that targeting the SPHK/S1P axis may provide an alternative to tyrosine kinase inhibitors, alone or in combination, for the treatment of aggressive mastocytosis and other hematological malignancies associated with the D816V-KIT mutation.

## Introduction

Sphingosine-1-phosphate (S1P) is a lipid mediator with multiple biological functions. Evidence indicates that S1P plays a significant role in cancer as it promotes cell migration and cell proliferation, prevents cell death, and induces angiogenesis (1, 2). S1P is generated in cells in response to growth factors, cytokines, and other extracellular stimuli through the activation of two isoforms of sphingosine kinase (SPHK1 and SPHK2) (3–5). Upregulated expression of SPHK, particularly SPHK1, and increased levels of S1P have been linked with poor cancer prognosis and resistance to cytotoxic therapy agents in malignant cells from solid tumors, lymphomas, and leukemias (1, 6–8). Selective targeting of either SPHK1 or SPHK2 can effectively inhibit malignant cell growth in culture and in xenograft models and the preferential effect of one isoform over the other appears to be cell type specific (1, 9–15). Nevertheless, an inhibitor of SPHK2, ABC294640, is in early clinical trials for patients with pancreatic cancer and diffuse large B cell lymphomas (clinical trials NCT01488513 and NCT02229981) (16). In addition, safingol, which inhibits SPHK1 and SPHK2, has been used in combination with cisplatin in Phase I clinical trials (clinical trial NCT00084812) for the treatment of locally advanced or metastatic solid tumors (17).

Abnormal mast cell (MC) proliferation and accumulation in various tissues occurs in mastocytosis, which in its more aggressive forms can be life-threatening (18, 19). This increased MC burden in systemic mastocytosis (SM) is most often associated with the presence of an activating mutation in the catalytic domain of KIT, the receptor for stem cell factor, that causes ligand-independent activation of KIT and downstream signaling, especially within the MC compartment (20–22). Thus, targeting KIT activity has been a focus of pharmacological strategies to treat aggressive SM, with the limitation that the extended tyrosine kinase conformation of D816V-KIT is not inhibited by tyrosine kinase inhibitors such as imatinib (21, 23). In addition, midostaurin (PKC412), which effectively blocks D816V-KIT activity (24) and the growth of MC expressing D816V-KIT (25), has time-limited effects on improvement of clinically relevant responses in patients with aggressive SM (26). Thus, new strategies that target critical growth signaling pathways in neoplastic MCs are needed.

Mast cells of murine or human origin express both SPHK1 and SPHK2 isoforms, which are activated following IgE receptor stimulation, and S1P consequently generated plays a critical role in IgE-mediated release of mediators and cytokines (27–31). In addition, receptors that are important for maturation/growth/survival of MCs such as KIT and the IL-3 receptor also stimulate SPHKs (27). However, it has not been studied whether SPHK and S1P regulate MC growth and survival.

Here, we show that SPHK1 and SPHK2 are upregulated in neoplastic human MCs and are important regulators of their proliferation and survival in culture. Inhibition of SPHK2 reduces the growth of primary mouse and human mast cells (HuMCs) cultures and neoplastic human MC lines by slowing their entry into the cell cycle. On the other hand, SPHK1 inhibition induces cell cycle arrest followed by cell death in neoplastic MCs expressing D816V-KIT but not in normal MCs. We demonstrate that this effect is mediated by a previously unrecognized regulation of the checkpoint kinases CHK1 and CHK2 and the activation of the DNA damage response (DDR) cascade. Inhibition of SPHKs also reduces the number of malignant MCs from bone marrow of mastocytosis patients *ex vivo* and in a preclinical mouse model of tumor xenografts using a MC line with D816V-KIT. Our results show promise for clinical investigation of this non-tyrosine kinase-based approach to the treatment of aggressive SM and other hematological malignancies with D816V-KIT.

## Materials and Methods

### Reagents

Reagents were obtained as follows: anti-rabbit IgG 800CW and anti-mouse IgG 680 RD from Licor Biosciences (Lincoln, NE, USA); goat anti-rabbit IgG-AF488 from Abcam (Cambridge, MA, USA); and anti-CD117-BV605, anti-FcεRI-BV421, and anti-CD25-BV711 from Biolegend (San Diego, CA, USA). Antibodies against phospho-AKT(Ser473), BCL-2-associated X protein (BAX), B-cell lymphoma (BCL2), cleaved caspase 2 (CASP2), caspase 3 (CASP3), cleaved CASP3, M-phase inducer phosphatase 3 (CDC25c), cyclin-dependent kinase 1 (CDK1), phospho-CHK2(Thr68), phospho-ERK(Thr202/Tyr204), p38, phospho-p38(Thr180/Tyr182), and p53 were from Cell Signaling Technology (Danvers, MA, USA); anti-CASP2 and anti-CHK2 were from Millipore (Billerica, MA, USA); anti-CHK1, anti-phospho-CHK1(Ser345), hIL-6, and hSCF were from R&D Systems (Minneapolis, MN, USA); anti-phospho-p53(Ser20) (human) and anti-GADD45 were from Santa Cruz (Santa Cruz, CA, USA); anti-H2A histone family member X (H2AX) and anti-phospho-H2AX (Ser130) were from Abclonal (Woburn, MA, USA); anti-SPHK1 was from ECM Biosciences (Versailles, KY, USA), anti-SPHK2 was from MyBioSource (San Diego, CA, USA); anti-CD34-APC and anti-AKT were from BD Biosciences (San Jose, CA, USA); anti-ERK and anti-SPHK2 (used for IHC) were from Thermo Fisher Scientific (Waltham, MA, USA); anti β-actin and anti-SPHK1 (used for IHC) were from Sigma Aldrich (St. Louis, MO, USA); mouse IL-3 and SCF were from Peprotech Inc. (Rocky Hill, NJ, USA); the SPHK1 inhibitor SKI-178 was from Millipore and the SPHK2 inhibitor ABC294640 was from MedKoo Biosciences (Morrisville, NC, USA).

### Human Samples, Cell Cultures, and Cell Lysates

CD34^+^ peripheral blood progenitors from human blood and bone marrow aspirates from patients with SM were obtained following informed consent under protocols approved by the NIAID Institutional Review Board (98-I-0027 and 02-I-0277). The characteristics of these patients are specified in Table S1 in Supplementary Material. Primary HuMC cultures were derived from CD34^+^ progenitors as described (32, 33); and mononuclear cells from marrow aspirates were separated in a Ficoll gradient and cultured for 5 days in StemPro media supplemented with 100 ng/mL SCF (34).

HMC-1.1 and HMC-1.2 were kindly provided by Dr. Butterfield at the Mayo Clinic. HMC-1 cells, LAD-2 cells, and murine bone marrow-derived mast cells (BMMCs) from *Sphk1*-, *Sphk2*-deficient, and WT mice were cultured as described (28, 35, 36). P815 mastocytoma murine cells with a mutation homologous to the human D816V mutation were from ATCC (Manassas, VA, USA). HCT116 cells with or without the D816V-KIT introduced by CRISPR were from Thermo Fisher Scientific. P815 and HCT116 cells were cultured as specified by the respective providers. Cell lysates for Western blots were prepared from 1 × 10^6^ cells and processed as described (37).

### Flow Cytometry Analysis of MCs From Bone Marrow Aspirates

Bone marrow cells cultured for 5 days as described above were washed once with PBS, resuspended in 200 µL PBS containing aqua live/dead stain (Thermo Fisher Scientific), and incubated in the dark for 20 min at room temperature. After washing with 3% FBS in PBS, cells were incubated with 50 µL of an antibody cocktail containing anti-CD117(KIT)-BV605, anti-FcεRI-BV421, anti-CD34 -APC, and anti-CD25-BV711 for 30 min at room temperature. Cells were then washed once, resuspended in 200 µL PBS, and analyzed by flow cytometry. Cells were gated based on forward/side scatter, single cells, and live cells. CD34^−^/CD25^+^ cells co-expressing CD117(KIT) and FcεRI were analyzed.

### Determination of Cell Growth

2.5 × 10^6^ LAD2 or murine BMMC were plated in T25 flasks at 0.5 × 10^6^ or 1 × 10^6^/mL, respectively, and 4 × 10^6^ HCT116 cells at 0.5 × 10^6^/mL. Cells were incubated with or without inhibitors at the indicated concentrations for 3 days or as indicated. For BMMCs, culture medium was changed after 7 days, and cell concentration was adjusted. Viable cells excluding trypan blue were then counted. Alternatively, 5 × 10^4^ HMC-1 or P815 cells were plated in 96-well plates at 0.5 × 10^6^ cells/mL with or without inhibitors. At the indicated times, an equal volume of 2× CyQuant^®^Direct detection reagent containing a green fluorescent nucleic acid stain for viable cells (Thermo Fisher Scientific) was added to the wells. After 1 h at 37°C, fluorescence was measured at 480/535 nm.

### Cell Cycle Analysis

LAD2 or HMC-1 cells (1 × 10^6^) were plated at 0.5 × 10^6^ cells/mL in 6-well plates and cultured with the SPHK inhibitors or vehicle for 3 days. Cells were then harvested and fixed in 70% ethanol at 4°C for 2 h. After washing twice with PBS, cells were stained with PI/RNase Staining Buffer (BD Pharmingen) and analyzed on a LSR II (BD Biosciences) flow cytometer for PI positive signal (Emission at 605 nm). Single cells were gated and PI signal visualized using histogram plots. Distribution of cells containing DNA characteristic G0-G1, S, and G2/M cell cycle phases was determined using FlowJo as described (38).

### Cell Death/Apoptosis Analysis

The annexin V FITC Apoptosis Detection Kit II (BD Pharmingen) was used following the manufacturer’s protocol. Cells (1 × 10^6^ cells/mL) were incubated with vehicle or the SPHK inhibitors for 3 days and stained with FITC-annexin V for 30 min in the dark on ice. After washing, 7-amino-actinomycin D (7-AAD), which binds DNA with high affinity but is excluded by intact cells, was added to differentiate early apoptosis from late apoptosis or other forms of cell death, and cells were analyzed by flow cytometry (LSR II BD). The percentage of early apoptotic cells was determined as the frequency of annexin V positive/7-AAD negative cells, while the percentage of all dead cells was determined as the frequency of the 7-AAD positive population (annexin V positive and negative).

### Expression of Cell Cycle Genes by Quantitative PCR Arrays

Total RNA was isolated from HMC-1.2 cells (3 × 10^6^) treated with vehicle or SPHK inhibitors for 24 h. RNA was converted into cDNA using the RT^2^ First-Strand Kit (Qiagen), and then used as a template to perform a quantitative RT^2^ Profiler PCR Array for Human Cell Cycle (Qiagen PAHS-020Z) to analyze the expression levels of 84 genes involved in regulation of cell cycle. The raw threshold cycle data were analyzed using SA Biosciences web-based tool,^1^ as instructed by the manufacturer. An ingenuity pathway analysis (IPA) program (Mountain View, CA, USA) was employed for pathway analysis using the fold changes between the SPHK1-I- or SPHK2-I-treated cells compared with vehicle-treated cells. Changes in expression of cyclin genes were confirmed by using specific TaqMan gene expression assays (Thermo Fisher Scientific).

### Immunocytochemistry

Human mast cells, LAD2, HMC-1.1, and HMC-1.2 cells (2 × 10^5^ cells/200 μL) were plated for 20 min in LabTek II Chamber Slides (LabTek, Rochester, NY, USA) coated with polylysine 0.01% (Sigma-Aldrich). Cells were fixed with 4% PFA (Affimetrix, Cleveland, OH, USA) for 20 min at 4°C and permeabilized with PBS + 0.2% TritonX-100 for 30 min. After blocking with PBS + 1% BSA + Fc blocking antibody (1:50) (eBioscience), cells were incubated with anti-SPHK1 or anti-SPHK2 for 1 h at 4°C with agitation and proteins detected with AF488-labeled secondary antibody. DAPI was used for nuclear staining in combination with Prolong Gold Antifade Mountant (Thermo Fisher Scientific). Images were captured with a Leica DMI 8 confocal microscope and analyzed with Imaris software.

### Sphingolipid Analysis

Lipids were extracted from 8 × 10^6^ HMC-1.2 cells following treatment with SPHK1-I (10 µM) or SPHK2-I (50 µM) for 16 h. Ceramides, sphingosine, and S1P were measured by HPLC-tandem MS by the Lipidomics Core at the Medical University of South Carolina on a Thermo Finnigan (Waltham, MA, USA) TSQ 7000 triple quadrupole mass spectrometer, operating in a multiple reaction-monitoring positive ionization mode as described (39). The sphingolipid concentration was normalized using inorganic phosphate measurements of the lipid extracts.

### Mice and Xenograft Model

All studies involving mice were performed in accordance with NIH guidelines and with the animal study proposal (LAD2E) approved by the NIH/NIAID Animal Care and Use Committee. WT, *Sphk1*-, and *Sphk2*-null mice were generated as described (40), and bred within NIAID animal facilities.

For the xenograft tumor model, NSG mice (NOD-*scid* IL2R γ^null^) from Jackson laboratories (Bar Harbor, ME, USA) (6–8 weeks) were injected subcutaneously with 1 × 10^6^ HMC-1.2 neoplastic MCs. Cells were washed and injected in 100 µL of RPMI medium into the right flank. Tumor size was measured with a Mitutoyo IP65 caliper. Tumor volume was calculated following the solid tumor formula: volume (mm^3^) = (length × width^2^)/2 (41). Once the tumors reached 50 mm^3^ (within 18–23 days), mice were injected i.p. daily for a maximum of 15 days with SPHK1-I or SPHK2-I at 20 or 40 mg/kg, as indicated, or vehicle (PEG400 with 5% DMSO) and the tumor size was measured after each injection. Mice were euthanized when tumors reached 1.5 cm in one dimension (days 11–15).

### Statistical Analysis

Statistical significance was determined using Student’s *t*-test. Comparison of changes over time between different groups was performed using two-way ANOVA using Prism software. A *p* value of less than 0.05 was considered significant. Data are shown as mean ± SEM unless specified otherwise.

## Results

### SPHKs Regulate the Growth of Normal Murine and Human MCs

**Figure 1:**
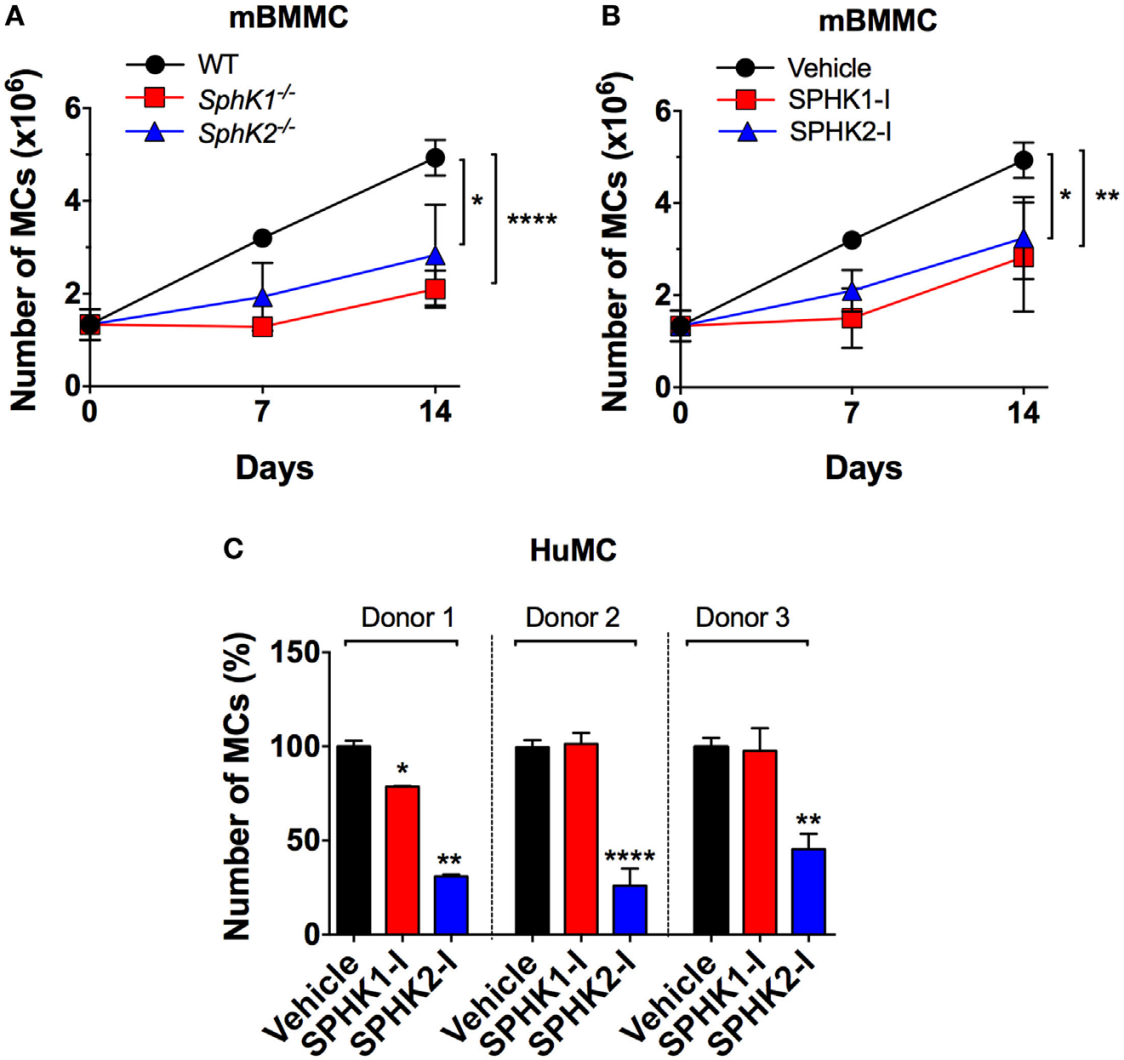
SPHK1 and SPHK2 are critical for the growth and survival of mouse and human MCs. **(A)** Number of viable bone marrow-derived mast cells (BMMCs) from WT, *Sphk1*-, and *SphK2*-deficient mice over time in culture. **(B)** Number of viable WT BMMCs treated with either the SPHK1-specific inhibitor SKI-178 (5 µM) (SPHK1-I) or the SPHK2-specific inhibitor ABC294640 (50 µM) (SPHK2-I). 0.5 × 10^6^ cells were plated and cultured for 14 days and viable cells excluding trypan blue counted at the indicated times. Values are mean ± SEM of data from three independent cultures. **(C)** Effect of SPHK-I in the growth of 5- to 6-week-old CD34^+^-derived human mast cells (HuMCs). Cultures derived from three independent healthy donors were incubated with SPHK1-I (10 µM), SPHK2-I (50 µM), or vehicle (0.1% DMSO) for 5 days and viable cells excluding trypan blue counted. Data are shown as mean ± SEM of triplicate wells and are representative of three independent experiments. Significant differences from untreated and treated pairs are shown thus; *p < 0.05, **p < 0.01, ***p < 0.001.

To investigate the role of SPHKs on MC proliferation, we first compared the growth rates of MCs derived from *Sphk1*- and *Sphk2*-deficient bone marrow progenitors (BMMC) to normal BMMCs. As shown in Figure 1A, growth rates of *Sphk*-deficient cells were significantly reduced compared with WT BMMCs. Treatment WT-BMMCs with small molecule inhibitors specific for SPHK1 [SKI-178 (42); referred here as SPHK1-I] or SPHK2 [ABC294640 (12, 43); referred in here as SPHK2-I] exhibited similar effects on growth rates as BMMCs genetically deficient in Sphk1 or Sphk2 (Figure 1B). We then explored the effect of these inhibitors on the proliferation/survival of 6-week cultures of CD34^+^-derived HuMCs containing more than 85% KIT^+^/FcεRI^+^ double-positive MCs. While SPHK1-I did not show any significant effect, treatment with SPHK2-I markedly reduced HuMC numbers in cultures from three different healthy donors (Figure 1C). The data support a role for SPHKs in the growth/survival of normal human and mouse MCs, although the relative involvement of SPHK1 appears to differ between human and mouse by mechanisms unrelated to the relative expression of SPHK1 since SPHK1 is more abundant in human than in mouse MCs (28).

### SPHKs Are Upregulated in Neoplastic MCs

**Figure 2:**
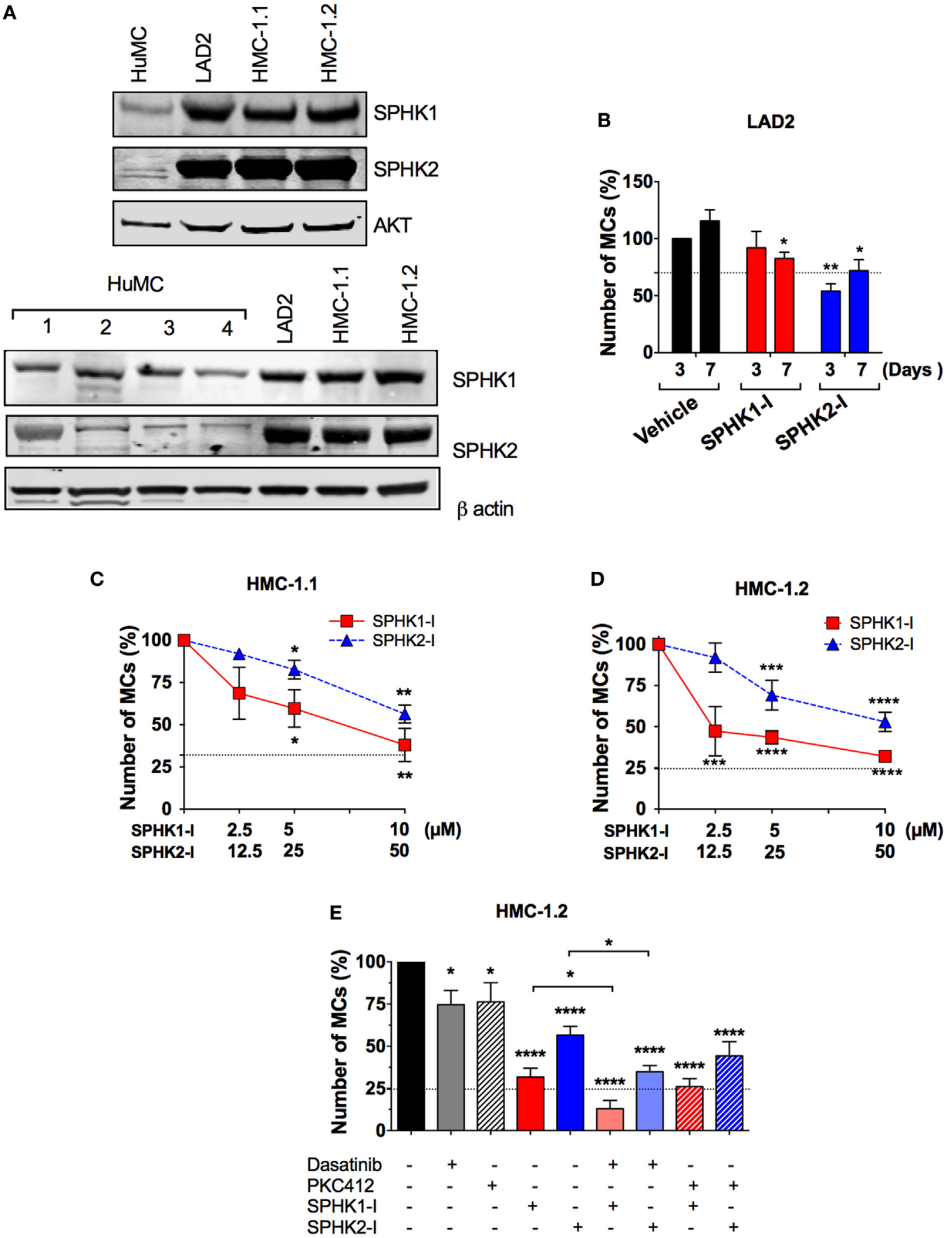
Enhanced expression of SPHK1 and SPHK2 in neoplastic human MCs and their effect on cell growth. **(A)** Representative Western blots from one of three independent experiments showing immune-reactive bands for SPHK1 (46 kDa) and SPHK2 (70 kDa) in human mast cells (HuMCs) derived from five different donors (upper and lower sets of blots) and the neoplastic MC lines LAD2, HMC-1.1, and HMC-1.2. Lysates were prepared from 1 × 10^6^ cells and equal protein loading obtained by adjusting to total AKT or β-actin levels. Donor 2 in Figure 1C corresponds to donor #2 in the lower set of blots. **(B)** Effect of SPHK1-I (5 µM) and SPHK2-I (50 µM) in the growth of LAD2 cells. Cells excluding trypan blue were counted 3–7 days after treatment with the inhibitors or vehicle. The values represent the percentage of viable cells respect to the number of vehicle-treated viable cells after 3 days in culture (first column). Data are represented as mean ± SEM of three independent experiments. Effect of the indicated concentrations of SPHK1-I and SPHK2-I in the growth HMC-1.1 **(C)** and HMC-1.2 **(D)** for 3 days. **(E)** Effect of SPHK inhibitors alone or in combination with KIT inhibitors on the growth of HMC-1.2 cells. SPHK1-I (5 µM) or SPHK2-I (50 µM) were added to cultures at day 0 alone or together with 300 nM midostaurin (PKC412) or 1 µM Dasatinib for 3 days. In panels **(C–E)**, Cyquant cell proliferation assay was used to assess growth inhibition, and data are represented as mean ± SEM of three independent experiments each performed in triplicate. The number of cells at the end of the experiment in untreated controls was considered 100%. Horizontal dotted lines represent the number of cells originally plated (as a percentage of the cell count at day 3 in untreated cultures). Significant differences from untreated and treated pairs are shown thus; *p < 0.05, **p < 0.01, ***p < 0.001.

Enhanced SPHK expression in a number of malignant cell types has been associated with increased proliferation, cancer prognosis, and clinicopathological parameters of disease (1, 44). Similarly, we found an increase in the expression of SPHK1 and SPHK2 in mastocytosis MC lines (i.e., LAD2 and HMC-1) compared with normal HuMCs (Figure 2A; Figure S1 in Supplementary Material), which was variable depending on the donor. Of note, expression and activity of SPHK2 in normal HuMCs albeit much lower than in neoplastic cells were on the average higher than that for SPHK1 (28).

Although SPHKs are predominantly cytosolic enzymes (45), both SPHK1 and SPHK2 (46, 47) can be abundantly expressed in the nucleus of cancer cell lines such as MCF7, COS7, and HeLa cells and a higher nuclear expression of SPHK1 has been associated with significantly shorter survival in breast cancer patients (44). Using IHC for SPHK1 and SPHK2, we observed that SPHK1 and SPHK2 were distributed homogeneously throughout the cell in normal HuMCs, and more concentrated in perinuclear areas and the plasma membrane (Figure S1 in Supplementary Material). Analysis of the confocal IHC images confirmed the increased overall cellular expression of SPHKs in neoplastic MCs and a higher density of SPHK, particularly SPHK1, in the nucleus of neoplastic MCs compared with normal HuMCs (Figure S1 in Supplementary Material). This was especially apparent in HMC-1.2 cells. However, the percentage of nuclear SPHK (respect to the total cellular content) was similar among the cell types suggesting that the enrichment of nuclear SPHK was the result of higher overall expression but not of a differential distribution pattern in neoplastic cells.

### SPHK1 Is Critical for the Growth/Survival of Cells With D816V-KIT

We then examined whether the growth of neoplastic MCs was influenced by SPHK1-I or SPHK2-I as we found in normal MCs. While treatment with SPHK2-I resulted in a 40–50% reduction in the number of both LAD2 (Figure 2B) and HMC-1 cells (Figures 2C,D), the effect of SPHK1-I differed depending on the MC line. Similar to normal HuMCs cultures (Figure 1C), SPHK1-I had only a modest inhibitory effect in the growth of LAD2 MCs (Figure 2B). However, unlike LAD2 cells, the growth of HMC-1.1 and HMC-1.2 cells carrying oncogenic KIT mutations was markedly reduced by 60% (Figures 2C,D). Furthermore, HMC-1.2 MCs, harboring the D816V-KIT mutation in addition to the G560V-KIT present in HMC-1.1, were significantly more sensitive to lower concentrations of SPHK1-I than HMC-1.1 cells (compare Figures 2C,D). This contrasted with the lack of effects of SPHK1-I on the growth of normal HuMCs (Figure 1C), although the effects varied slightly in the different donors, potentially reflecting, to some extent, differences in SPHK expression.

Of note, SPHK1 and 2 inhibitors were more effective at reducing the expansion of HMC-1.2 cells than 300 nM PKC412 or 2 µM dasatinib (Figure 2E), which are known to inhibit D816V-KIT. Combination treatment with dasatinib or PKC412 and SPHK inhibitors showed higher inhibition than either inhibitor alone, although the combination with PKC412 was not statistically different compared to SPHK inhibitors alone (Figure 2E).

We also examined whether other cells carrying D816V-KIT also had higher sensitivity to SPHK1-I. The growth of the mastocytoma MC line P815 containing the murine equivalent to human D816V-KIT was also remarkably inhibited by SPHK1-I compared with normal BMMC (Figure S2A in Supplementary Material). Furthermore, the colon cancer epithelial cell line HCT116 was susceptible, as most cancer cells are, to growth inhibition by either SPHK1-I or SPHK2-I, but they were even more susceptible earlier on to the effects of the SPHK1-I when a mutation in D816V-KIT was additionally introduced (Figure S2B in Supplementary Material). The finding of a higher susceptibility of cells carrying D816V-KIT to SPHK1-I is of interest given that patients with these mutations are less responsive to KIT inhibitor treatments.

### Differential Effects of SPHK1 and SPHK2 Inhibition on Cell Cycle Arrest and Survival of Neoplastic MCs

**Figure 3:**
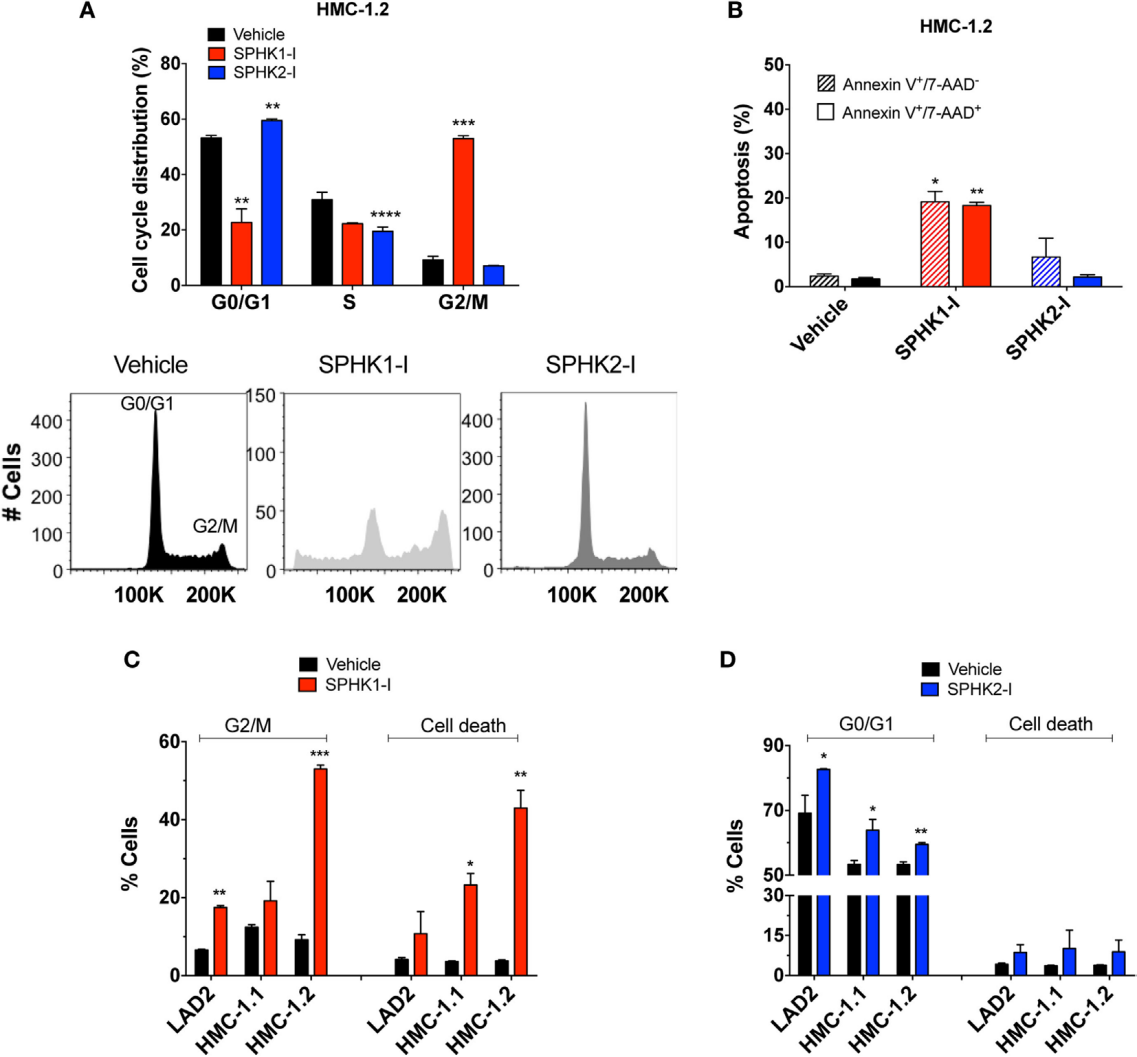
Effect of SPHK1 or SPHK2 inhibition on cell cycle arrest and cell death in human neoplastic mast cells. **(A)** Cell cycle analysis distribution of PI-stained HMC-1.2 cells treated with SPHK1-I (5 µM), SPHK2-I (50 µM), or vehicle (0.1% DMSO) for 3 days. After 3 days of treatment, cells were stained with propidium iodide (PI) and analyzed by flow cytometry. Percentage of cells in the various phases of the cell cycle is represented in histogram form. A representative cell cycle distribution by FACS analysis is also shown. **(B)** Inhibition of SPHK1 causes cell death in HMC-1.2 cells. HMC-1.2 treated with SPHK inhibitors as in panel **(A)** were stained with 7-AAD and annexin V. Annexin V^+^/7^−^AAD^−^ cells were considered as cells in early apoptosis (hatched bars) while annexin V^+^/7^-^AAD^+^ cells (black bars) were considered as cells in late apoptosis or other possible types of cell death. Cells that stained only for 7-AAD were in a negligible proportion. Comparison of the effects of SPHK1-I **(C)** and SPHK2-I **(D)** in cell cycle distribution and apoptosis in the different neoplastic MC lines. Cell cycle distribution was determined as in panel **(A)** and cell death as in panel **(B)**, except that the numbers denote the combined percentage of annexin V^+^/7^−^AAD^−^ and annexin V^+^/7^−^AAD^+^. In panels **(A–D)**, the values are the mean ± SEM of three separate experiments. Significant differences from untreated and treated pairs are shown thus; *p < 0.05, **p < 0.01, ***p < 0.001.

We next explored how SPHK1-I and SPHK2-I affected cell growth of neoplastic MCs, focusing on HMC-1.2 since these cells have the D816V mutation commonly found in SM. In HMC-1.2, inhibition of SPHK1 caused a prominent cell cycle arrest in G2/M (Figure 3A) accompanied by extensive apoptosis evidenced by annexin V^+^ staining (Figure 3B), while in contrast inhibition of SPHK2 resulted in the accumulation of cells in the G0/G1 phases and reduction of the percentage of cells in the S phase (Figure 3A) with no major effects on cell death (Figure 3B). G2/M arrest and apoptosis induced by SPHK1-I were significantly more prominent in HMC-1.2 than in HMC-1.1 and LAD2 cells (Figure 3C), in agreement with their overall effects on cell numbers. SPHK2-I, however, prevented the exit from G1 into the S phase in a comparable manner in all neoplastic MCs (Figure 3D), consistent with its similar effects reducing cell numbers in all the cell lines. Treatment of HMC-1.2 with either of these inhibitors did not significantly affect the expression of SPHK1 or SPHK2 (data not shown).

Subsequent studies on the mechanisms by which these inhibitors regulate growth and survival were mostly performed in neoplastic MCs with D816V-KIT, i.e., HMC-1.2, because of the clinical relevance of this mutation and its resistance to most tyrosine kinase inhibitors.

### Effect of SPHK1 and SPHK2 Inhibitors on Sphingolipid Metabolites

**Figure 4:**
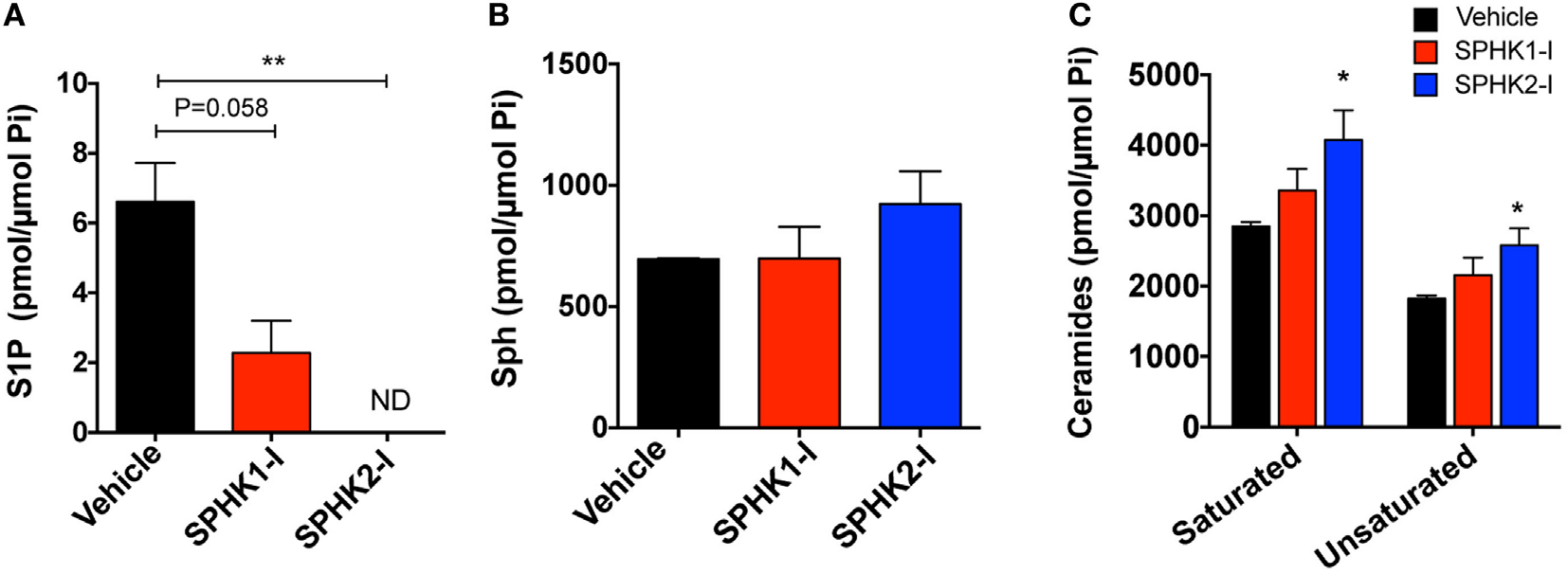
Effects of SPHK1 and SPHK2 inhibition in the intracellular levels of sphingolipid metabolites. HMC-1.2 cells were cultured in the presence of SPHK1-I (5 µM), SPHK2-I (50 µM), or vehicle (0.1% DMSO) overnight. Cellular lipids were extracted and the cellular amounts of sphingosine-1-phosphate (S1P) **(A)**, sphingosine (Sph) **(B)**, and ceramide **(C)** determined by mass spectrometry. The amount of all the saturated (C14, C16, C18, C20, C22, and C26) and unsaturated (C18:1, C20:1, C20:4, C22:1, C24:1, and C26:1) ceramides were combined for simplicity. The total content of phospholipid per sample was used to normalize the results. The most abundant ceramide species were C24 and C24:1. The data are presented as mean ± SD of two separate experiments each performed in duplicates. Significant differences from untreated and treated pairs are shown thus; *p < 0.05, **p < 0.01. ND indicates “not detected.”

As expected, inhibition of SPHK1 or SPHK2 caused substantial reductions in the cellular concentration of S1P, the product of these lipid enzymes (Figure 4A). The reduction in S1P was particularly notable with SPHK2-I treatment (Figure 4A) and was accompanied by some accumulation of the substrate, sphingosine (Figure 4B). Since sphingosine can be converted into ceramide and, in contrast to S1P, ceramide is a sphingolipid metabolite that mediates pro-apoptotic and anti-growth effects (7, 48), we thus examined whether cell death induced by SPHK1-I could be related to increases in ceramide levels. However, increases in ceramides at the indicated times were only significantly detected in cells treated with SPHK2-I (Figure 4C) and therefore, the distinct apoptosis induced by SPHK1-I does not seem to implicate ceramide production. Thus, both inhibitors reduced S1P levels in these cells but only SPHK2-I resulted in detectable increases in ceramides that could contribute to SPHK2-I-mediated growth inhibition, albeit not substantially to apoptosis.

### SPHK1-I and SPHK2-I Differentially Affect the Expression of Genes Regulating the DDR and Repair Pathways

**Figure 5:**
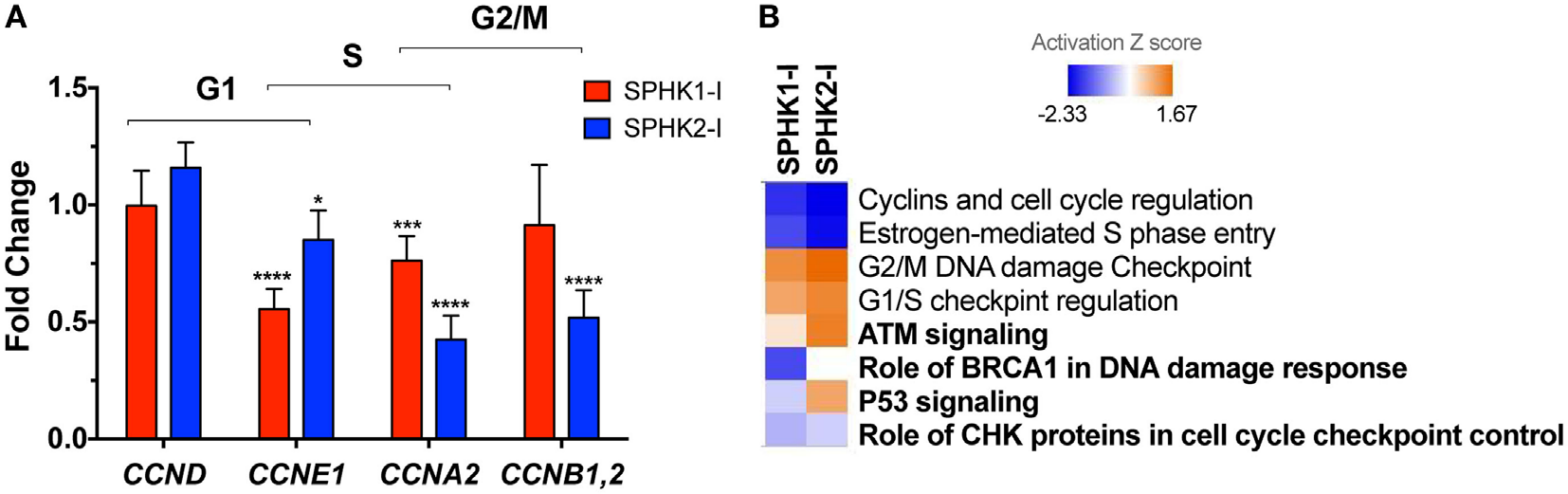
Effects of SPHK1 and SPHK2 inhibition in the expression of cell cycle genes. **(A)** Relative changes in the mRNA levels of the indicated cyclins in HMC-1.2 after 24 h treatment with 5 µM SPHK1-I or 50 µM SPHK2-I. Changes in the expression of cyclins were initially determined using a RT^2^ Profiler PCR Array and confirmed by Taqman gene expression assays. **(B)** Heat map of the canonical pathways affected by the treatment of HMC-1.2 with 5 µM SPHK1-I or 50 µM SPHK2-I for 24 h. Changes in expression of 84 genes involved in the cell cycle induced by SPHK1-I or SPHK2-I treatment were determined using a RT^2^ Profiler PCR Array. Fold changes in the genes induced by either inhibitor were then analyzed using the ingenuity pathway analysis (IPA) with 1.5-fold as a cutoff. Shown is a comparison analysis done by IPA of the canonical pathways most significantly affected by either inhibitor and sorted by the activation *Z* score (blue: predicted inhibited and red: predicted activated). In bold, pathways related to DNA damage response cascade.

To gain insights into the mechanisms of growth inhibition and induction of apoptosis in HMC-1.2 cells by SPHK1-I, we investigated the effects of these inhibitors on the expression of 84 genes that are critical for cell cycle regulation using a qPCR array. The original datasets with the changes in gene expression induced by SPHK1 or SPHK2 inhibitors can be found in NCBI’s Gene Expression Omnibus (49) and are accessible through GEO Series accession number GSE111060.^2^ Treatment of HMC-1.2 cells with either SPHK1-I or SPHK2-I reduced cyclin E (*CCNE1*) mRNA expression, a cyclin responsible for late G1 or G1/S transition. Expression of cyclins A and B, which facilitate progression into the S and G2 phases (50), was also reduced by the inhibitors although more significantly so by SPHK2-I (Figure 5A). Analysis of the expression of all cell cycle-related genes in the array with the IPA software predicted a downregulation of “*S phase entry*” and increased “*G1/S and G2/M checkpoint regulation*” as Canonical Pathways regulated by both SPHK1-I and SPHK2-I (Figure 5B; Table S2 in Supplementary Material). Surprisingly, these results suggest that both inhibitors similarly restrict progression into S phase and imply that the accumulation in G2/M observed only after SPHK1-I treatment occurs by mechanisms other than a specific downregulation of cyclins regulating transitions into G2 or mitosis.

As shown in Figure 5B and Table S2 in Supplementary Material, analysis by IPA of all cell cycle genes affected by the inhibitors gave further insights into these potential mechanisms. IPA suggested that pathways related to DDR mechanisms (noted in bold in Figure 5B) were distinctly regulated by these inhibitors. As pathways involved in the DDR are key in coordinating cell cycle progression with DNA repair and cell death/survival decisions, this analysis raised the possibility that arrest on G2/M by SPHK1-I treatment could be the result of unrepairable DNA damage or incomplete replication instead of regulation of cyclin expression.

### Inhibition of SPHK1 Causes CHK2-Mediated DDR Cascades Leading to Cell Death in HMC-1.2 Cells

**Figure 6:**
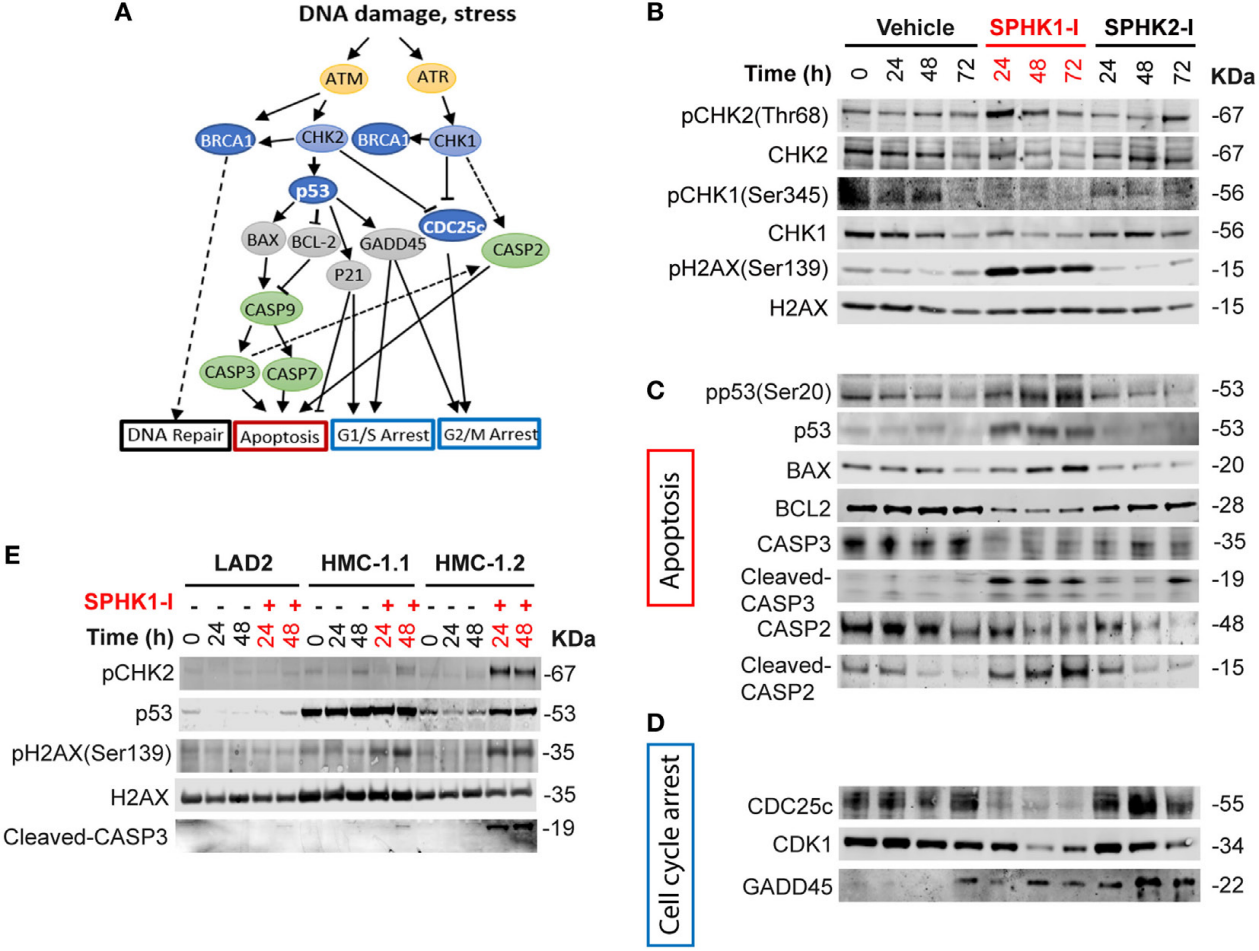
Activation of the DNA damage response (DDR) cascade by inhibition of SPHK1. **(A)** Schematic representation of players in the DDR cascade. **(B–D)** Western blots analysis of HMC-1.2 lysates after treatment with SPHK1-I (10 µM), SPHK2-I (50 µM), or vehicle (0.1% DMSO) for 24, 48, or 72 h, showing changes in the activation/levels of various effectors in the DDR. **(C)** CHK2-dependent effectors of the DDR involved in the apoptotic pathway as illustrated in panel **(A)**. **(D)** CHK2-dependent DDR effectors involved in cell cycle arrest as illustrated in panel **(A)**. **(E)** Western blot analysis showing the phosphorylation state or expression levels of the indicated DDR effectors after the treatment of LAD2, HMC-1.1, and HMC-1.2 cells with 10 µM SPHK1-I or vehicle for 24 and 48 h. All blots are from a representative experiment of at least three separate experiments. Each membrane was probed with β-actin to demonstrate equal loading and these blots are shown in Figures S3A and S6 in Supplementary Material.

We next investigated whether the DDR-related pathways were indeed affected by inhibition of SPHK1 in HMC-1.2 cells and which of the possible DDR cascades were involved. DNA damage is sensed by the PI3K-like kinases ataxia telangiectasia and Rad3-related protein (ATR) and ataxia telangiectasia mutated (ATM). ATR and ATM phosphorylate CHK1 and CHK2, respectively, which coordinate signaling cascades leading to cell cycle arrest and DNA repair or induce cell death or senescence if the damage cannot be repaired (51–53) (Figure 6A). While no significant effect on CHK1 or CHK2 phosphorylation was observed in cells treated with SPHK2-I, treatment with SPHK1-I markedly increased the phosphorylation of CHK2 in Thr68 (Figure 6B; Figure S3 in Supplementary Material), a site phosphorylated by ATM, suggesting an activation of ATM in response to double-stranded breaks or telomere damage (54, 55). Consistent with this conclusion, HMC-1.2 cells treated with SPHK1-I showed increased phosphorylation of H2AX at Ser139 an early marker of double-stranded breaks. By contrast, SPHK1-I did not affect the phosphorylation in Ser345 of CHK1 but reduced CHK1 expression, indicating a unique activation of the DDR in HMC-1.2 cells, with CHK2 activation but simultaneous depletion of CHK-1, which could have catastrophic effects on cells.

Phosphorylation of CHK2 may activate an array of effectors in the DDR that orchestrates the cell outcome (Figure 6A). Phosphorylation of p53 at Ser20, a residue that is a substrate for CHK2, was substantially increased by SPHK1-I. Increased p53 phosphorylation, as expected, resulted in the stabilization of p53 and thus increased levels compared with untreated cells (Figure 6C; Figure S3 in Supplementary Material). Downstream of p53 and consistently with its tumor suppressor activity, the levels of its transcriptional targets, the pro-apoptotic BAX, and the anti-apoptotic BCL2 were upregulated and downregulated, respectively. As a consequence, the executioner CASP3 was cleaved and activated. Cleaved CASP2 was also increased after treatment with SPHK1-I but not with SPHK2-I (Figure 6C; Figure S3 in Supplementary Material). Although CASP2 cleavage may occur *via* CASP3, CASP2 activation by SPHK1-I is also in agreement with the observed reduction in CHK1 levels, an effect that has been reported to limit DNA repair and induce apoptosis *via* CASP2 (56–58) (Figure 6A). The results suggest that inhibition of SPHK1 causes cell death in HMC-1.2 by selectively activating the CHK2/p53/CASP3 pathway and causing CHK1 depletion with consequent CASP2 activation.

Activation of the DDR may also lead to arrest of cells in various stages of the cell cycle. Consistent with the observed cell cycle arrest in G2/M, SPHK1-I but not SPHK2-I caused a depletion of the dual-specificity phosphatase CDC25c (Figure 6D; Figure S3 in Supplementary Material) which removes the inhibitory phosphate residues from CDK1 and allows the cell cycle to proceed. This effect on CDC25c is likely to be a consequence of SPHK-1 induced activation of CHK2 since CHK2 was reported to mediate either ubiquitination/degradation or phosphorylation/inactivation of CDC25c (53, 55). In addition, CDK1 expression levels were also markedly reduced by SPHK1-I treatment. The depletion of CDK1 ensures no further progression into mitosis. Furthermore, the expression of growth arrest and DNA damage-inducible (GADD45α), another transcriptional target of p53 that mediate cell cycle arrest (59), were increased in HMC-1.2 cells treated with either SPHK1-I or SPHK2-I (Figure 6D; Figure S3 in Supplementary Material). Overall, the data showing GADD45α upregulation supports the view that both inhibitors slow down entry into the cell cycle (G1/S) but an additional and more prominent and catastrophic consequence of SPHK1 inhibition in HMC-1.2 cells is the activation of the DDR, with CHK2-mediated G2/M arrest *via* CDC25c and CDK1 depletion as well as apoptotic cell death *via* CHK2/p53 and CHK1/CASP2 pathways.

### Activation of CHK2 by SPHK1-I Is More Prominent in Cells With D816V-KIT

We then tested whether the activation of this pathway by SPHK1-I correlated with its effects on the cell cycle and apoptosis in the different cell lines. Concomitantly with the effect on G2/M arrest and apoptosis, the effect of SPHK1-I treatment on CHK2 phosphorylation and CASP3 activation was more pronounced in HMC-1.2 than in HMC-1.1 and in LAD2 cells (Figure 6E; Figure S4 in Supplementary Material), which lack D816V-KIT. Similarly, treatment of murine P815 cells (harboring D816V-KIT) with SPHK1-I also induced Chk2 phosphorylation, p53 stabilization, and Casp3 activation, while this pathway was not observed in normal mouse BMMCs (Figure S5A in Supplementary Material). In addition, SPHK1-I induced a more pronounced CHK2 phosphorylation in HCT116 carrying D816V-KIT than the parental HCT116 cells (Figure S5B in Supplementary Material). Thus, these data implicate activation of the DDR by SPHK1-I as a mechanism involved in the increased susceptibility of cells with D816V-KIT to this inhibitor.

### SPHK Inhibitors Reduce Growth of Bone Marrow Neoplastic MCs From Patients and of Ectopic MC Tumors in Mice

**Figure 7:**
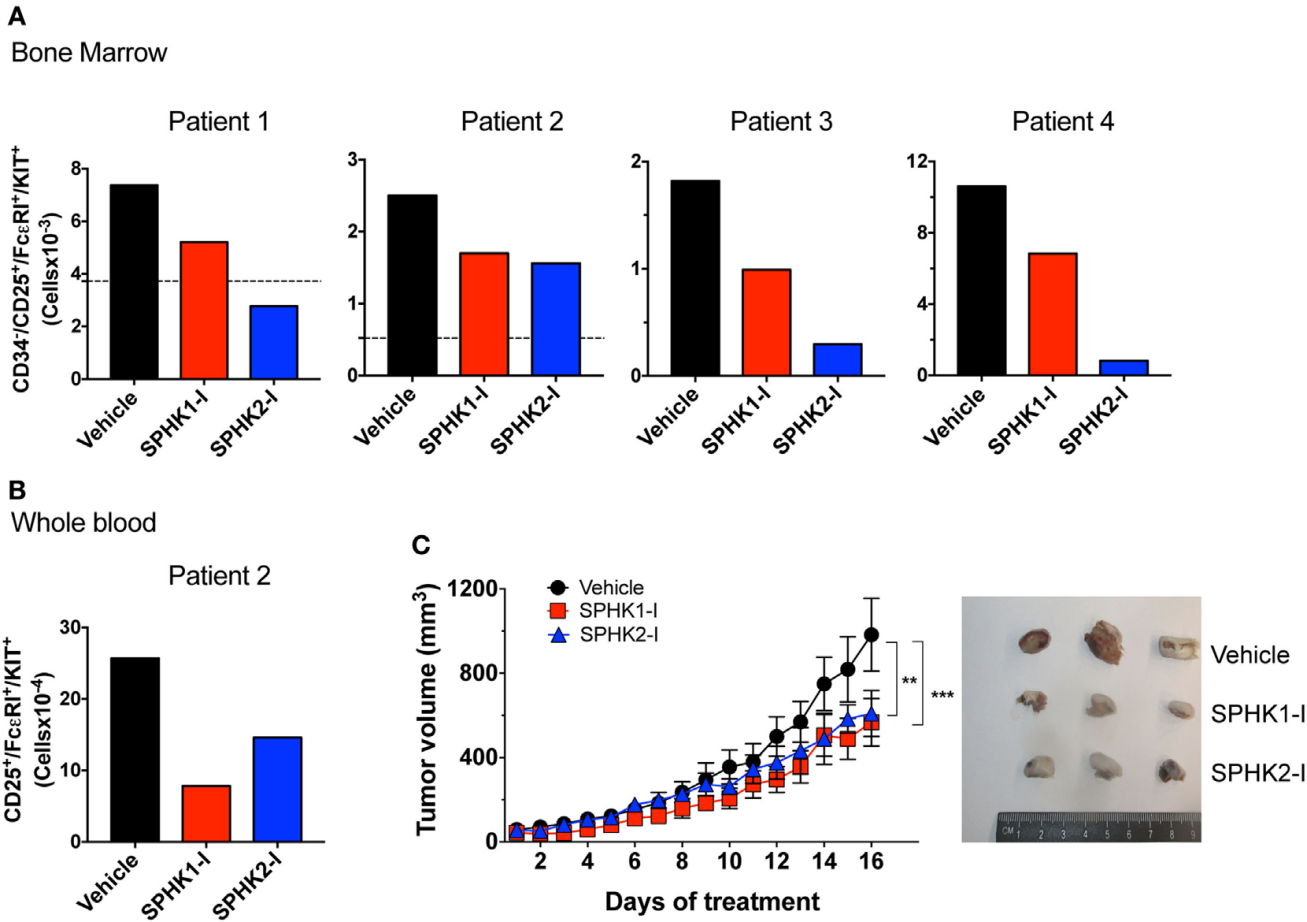
Inhibition of SPHK1 or SPHK2 reduces neoplastic mast cell (MC) number from bone marrows of patients with systemic mastocytosis and reduces growth of D816V-KIT MCs in a xenograft mouse model. **(A)** 0.5 × 10^6^ CD34^+^ cells from bone marrow aspirates from four independent donors with systemic mastocytosis (Table S1 in Supplementary Material) were cultured for 5 days in the presence of SPHK1-I (10 µM), SPHK2-I (50 µM), or vehicle (0.1% DMSO) in culture media. Total numbers of CD34^−^/CD25^+^/KIT^+^/FcεRI^+^ in each sample as determined by FACS analysis were calculated from the percentages of each gated population. Horizontal dotted lines represent the number of CD34^−^/CD25^+^/KIT^+^/FcεRI^+^ originally plated at day 0. This analysis at day 0 was not done for patients 3 and 4 due to limited number of available cells in those marrow aspirates. **(B)** CD34^+^ progenitors from whole blood were cultured for 6 weeks and treated with the inhibitors or vehicle as in panel **(A)** and analyzed by flow cytometry. Data were calculated as in panel **(A)**. **(C)** Mice were inoculated subcutaneously with 1 × 10^6^ HMC-1.2 cells and allowed for tumors to grow to 50 mm^3^ in volume. Mice were then injected daily (i.p.) with 20 mg/kg SPHK1-I, 40 mg/kg SPHK2-I, or vehicle (PEG400 + 5% DMSO) and tumor volume measured (*n* = 5/group). Shown on the right are images of three representative tumors excised from each group of mice.

To further assess the potential effectiveness of these inhibitors in preclinical models, we first treated bone marrow cells from four different patients with indolent and smoldering mastocytosis (see Table S1 in Supplementary Material) with SPHK1-1 or SPHK2-I. After 5 days in culture, the number of bone marrow malignant CD25^+^ MCs was substantially reduced by treatment with SPHK1-I and SPHK2-I (Figure 7A). Similarly, these inhibitors reduced by >50% the number of CD25^+^ MCs in 6-week-old CD34^+^-derived MC cultures from peripheral blood from one of the patients (Figure 7B). Whereas SPHK2-I was similarly effective in reducing MC numbers from normal donors (compare Figure 7B to Figure 1C), SPHK1-I significantly inhibited the growth of patient’s CD25^+^ MCs but not normal donor’s HuMCs (compare Figures 7B and 1C).

We next examined whether SPHK inhibition could also reduce the growth of D816V-KIT MCs *in vivo* in a xenograft mouse model. HMC-1.2 cells inoculated subcutaneously into immunodeficient mice formed palpable ectopic tumors that grew steadily over the course of the experiment. Mice treated daily with SPHKI-1 (20 mg/kg) or SPHK2-I (40 mg/kg) after the tumors were palpable (50 mm^3^) showed a significant reduction in tumor volume over time compared with those in mice treated with vehicle (Figure 7C). The inhibition of growth by SPHK1-I and SPHK2-I of primary neoplastic MCs from patients and of D816V-KIT MCs *in vivo* support the conclusion that SPHK inhibitors are appropriate candidates to consider for clinical applications in the treatment of mastocytosis.

## Discussion

Advanced SM can result in aggressive neoplastic MC infiltrates in tissues and end-of-organ compromise, requiring cytoreductive therapy. Current treatments targeting oncogenic D816V-KIT have shown limited long-term improvement and thus additional therapeutic approaches are needed. In this study, we find that SPHK1 and SPHK2, the enzymes responsible for the generation of the lipid mediator S1P, are upregulated in neoplastic human MCs and critically regulate their proliferation/survival. We also demonstrate the effectiveness of inhibition of SPHK1 and SPHK2 in reducing the numbers of malignant bone marrow MCs from patients with SM and *in vivo* in xenograft models. Of particular interest, inhibition of SPHK1 by SKI-178 prevented entry into mitosis and induced cell death most prominently in MCs with D816V-KIT. This involved the activation of CHK2 kinase concomitant with the depletion of CHK1, and downstream induction of the DDR leading to cell death. Overall, the data presented suggest great potential for SPHK inhibitors as alternatives to tyrosine kinase inhibitors in the treatment of aggressive SM.

Sphingosine kinases, especially SPHK1, are well-recognized regulators of proliferation and survival, although their involvement in these processes depends on the cell type. We find that neoplastic human MCs with oncogenic KIT mutations (including HMC-1.2 and bone marrow CD25^+^ malignant MC from patients with SM) as well as other cells carrying D816V-KIT, are more vulnerable to inhibition of SPHK1 than normal MCs or other cell counterparts lacking this mutation. This suggests that SPHK1 may mediate critical oncogenic pathways of D816V-KIT and, together with findings that loss of SPHK1 reduces bioactive mediator release by human MCs (28, 29), make SPHK1 a more desirable target for therapeutic development than SPHK2 since it blocks both proliferation and function of MCs. Nonetheless, the SPHK2-I used in this study may also represent a drug of interest in the treatment of aggressive mastocytosis since it is already in clinical trials for other malignancies and as demonstrated here, is also quite effective in reducing the numbers of CD25^+^ MCs and other neoplastic MCs. The finding that SPHK2-I also affects the growth of normal MCs may not detract from its use, since imatinib-induced depletion of normal MCs during imatinib therapy did not show adverse clinical manifestations (60).

An unexpected observation in this study was the differential effects of SPHK1 compared with SPHK2 inhibition despite their similar effects in reducing cellular S1P levels. SPHK1 inhibition resulted predominantly in G2/M arrest and an induction of apoptosis that could not be explained by increased ceramide levels, while SPHK2 inhibition accumulated cells in G0/G1, slowing entry into the cell cycle. The arrest in G0/G1 by blockage of SPHK2 is relatively consistent among cells (46, 61, 62) but reports suggest that blockage of SPHK1 activity may arrest cells in G2/M or G0/G1 depending on the cell type (14, 15, 63, 64). We found that both inhibitors similarly regulate the expression of cyclins in HMC-1.2, with a pattern consistent with a predicted arrest in G0/G1. A defining effect of SPHK1 suppression in neoplastic MCs, particularly those harboring D816V-KIT, was a deregulation of the DDR cascade which prevented entry into mitosis (G2/M arrest) of those cells that had passed the G0/G1 checkpoint, leading to them to programmed cell death. We propose this mechanism may be common to certain types of cancer cells and may partially explain the diverse effects on the cell cycle by SPHK1 blockage.

The activation of the DDR by SPHK1-I in D816V-KIT MCs was initiated by double-stranded breaks and induction of CHK2 activity. This effect has not been previously reported, but in other cancer cells, knockdown of SPHK1 and reduction in S1P levels sensitize them to DNA damage by ionizing radiation (IR) (65) or by doxorubicin (66). In D816V-KIT MCs, activation of CHK2 by SPHK1-I mediated a depletion of CDC25c, a phosphatase needed for the removal of an inhibitory residue in CDK1 before entry into mitosis, and a downregulation in CDK1, which is needed for the induction of mitosis when complexed with cyclin B. Activated CHK2 also phosphorylated and stabilized p53, which then increased BAX but reduced BCL2 levels, resulting in cleavage and activation of the executioner CASP3. In addition to CHK2 activation, CHK1 was distinctly depleted by SPHK1-I treatment, an observation consistent with a compromise in DNA repair and activation of CASP2. Inactivation or loss of CHK1 under robust genotoxic insults or IR damage drives a type of cell death that can bypass p53 deficiency. This has been the basis for recent treatment strategies in refractory cancers that would combine DNA damage-promoting drugs with inhibitors of CHK1 (57, 67, 68). Our data pose the intriguing possibility that SPHK1 inhibition on its own can act in D816V-KIT MCs like such proposed combination therapy (67) since it activates a CHK2-induced DDR while shutting down CHK1-induced repair, ensuring apoptosis of the transformed cells by p53-dependent and -independent mechanisms. These observations in turn suggest that synergistic treatments with anticancer drugs that cause DNA damage may prove even more effective.

The specific mechanism by which inhibition of SPHK1 by SKI-178 causes DNA double-stranded breaks is, however, unclear. The possibility that the inhibitor directly causes DNA damage is unlikely because LAD2 cells were not sensitive to this inhibitor or exhibited H2AX phosphorylation. A similar cascade of CHK2 activation, CDC25c depletion, p53 and CASP3 activation has been described after loss of telomere stability initiated by either reduced telomerase activity or alterations in the complexes that protect telomeres (55, 69–74). Since nuclear S1P generated by SPHKs stabilizes the catalytic subunit of telomerase (human telomerase reverse transcriptase, hTERT) (75), an interesting possibility is that SPHK1 may become critical for the proliferation and viability of cells with oncogenic D816V-KIT by preventing double-stranded breaks at fragile sites, particularly telomeres, during oncogene-induced replication stress. SPHK1 in these cells may support hTERT activity, which is increased in HMC-1.2 compared with normal human MCs (76), or promote repair mechanisms, which are upregulated in oncogenic cells. Nevertheless, further studies are needed to identify whether these or other mechanisms are involved.

In conclusion, this study determines that SPHK activities are important regulators for the proliferation of normal and neoplastic MCs *in vitro* and in MCs of patients with mastocytosis *ex vivo*. Moreover, we show that inhibition of their activity reduces MC burden in a xenograft model of neoplastic MCs and identify SPHK1 as an efficacious target for oncogenic KIT signaling. SPHK1 inhibition triggers an apoptotic program that resembles DDR-mediated apoptosis resulting from loss of telomere stability. Although more studies are necessary to evaluate the clinical potential of these inhibitors, our data show promise targeting SPHKs as an effective therapy, perhaps in combination with existing therapies, in the treatment of aggressive mastocytosis and other diseases involving D816V-KIT mutations.

## Ethics Statement

This study was carried out in accordance with the recommendations of the NIH and NIAID Institutional Review Board with written informed consent from all subjects. All subjects gave written informed consent in accordance with the Declaration of Helsinki. The protocols 98-I-0027 and 02-I-0277 were approved by the NIAID Institutional Review Board. This study was carried out in accordance with the recommendations of NIH guidelines and the NIH/NIAID Animal Care and Use Committee. The protocol was approved by the NIH/NIAID Animal Care and Use Committee (animal study proposal LAD2E).

## Author Contributions

RM-C initiated the study; GB, RM-C, and AT contributed equally to the bulk of the experimental work in this study; GB, RM-C, AT, AD, and YY performed experiments and analyzed data; HK scheduled patients’ visits and provided patients’ bone marrow aspirates; DM contributed to the design, intellectual content, and writing of the manuscript; AO designed and supervised the study, performed experiments, analyzed data, and drafted the manuscript. All authors critically revised and approved the manuscript.

## Conflict of Interest Statement

The authors declare that the research was conducted in the absence of any commercial or financial relationships that could be construed as a potential conflict of interest.

## Footnotes

^1^www.sabiosciences.com/pcrarraydataanalysis.

^2^https://www.ncbi.nlm.nih.gov/geo/query/acc.cgi?acc=GSE111060 (Accessed: February 23, 2019).

## Abbreviations

ATM, ataxia telangiectasia mutated; ATR, ataxia telangiectasia and Rad3-related protein; BAX, BCL-2-associated X protein; BCL2, B-cell lymphoma 2; BMMCs, bone marrow-derived mast cells; CASP2, caspase 2; CASP3, caspase 3; CDC25c, M-phase inducer phosphatase 3; CDK1, cyclin-dependent kinase 1; CHK, checkpoint kinase; DDR, DNA damage response; GADD45α, growth arrest and DNA-damage-inducible protein alpha; H2AX, H2A histone family member X; HuMC, human mast cell; IPA, ingenuity pathway analysis; KIT, stem cell factor receptor; MC, mast cell; p53, tumor protein p53; SM, systemic mastocytosis; S1P, sphingosine-1-phosphate; SPHK, sphingosine kinase; SPHK1-I, sphingosine kinase 1 inhibitor SKI-178; SPHK2-I, sphingosine kinase 2 inhibitor ABC294640.
