# Supplementary material for: Targeting Sphingosine Kinase Isoforms Effectively Reduces Growth and Survival of Neoplastic Mast Cells With D816V-KIT
Source: Front Immunol. 2018 Mar 28;9:631. doi: 10.3389/fimmu.2018.00631 (PMC5883065; doi:10.3389/fimmu.2018.00631)
Supplement: Supplementary file 1 [file data_sheet_1.PDF]

Supplementary Material for:

Targeting Sphingosine Kinase Isoforms Effectively Reduces Growth and Survival of Neoplastic Mast Cells with D816V-KIT

Geethani Bandara[1]†, Rosa Muñoz-Cano[2]†, Araceli Tobío[1]†, Yuzhi Yin[1], Hirsh Komarow[1], Avanti Desai[1], Dean D Metcalfe[1] and Ana Olivera[1]*
†These authors contributed equally

Supplementary Figures:

Figure S1
Figure S2
Figure S3
Figure S4
Figure S5
Figure S6

Supplementary Tables:

Supplementary Table I
Supplementary Table II

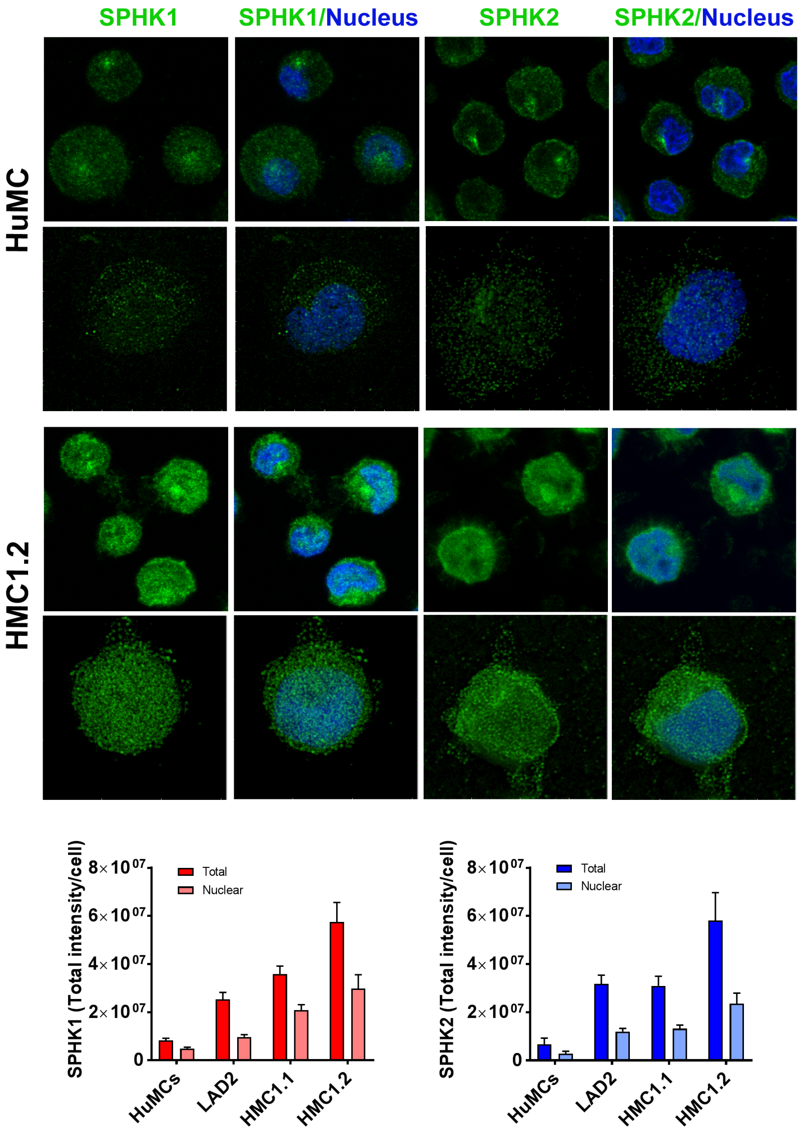

Figure S1- Subcellular distribution of SPHK1 and SPHK2 in normal HuMCs and neoplastic HMC-1.2 MCs. Confocal images obtained with a Leica DMI8 microscope (63X), of CD34^{+}-derived HuMCs and HMC-1.2 cells immune-stained to detect SPHK1 and SPHK2 (in green). Nuclei were stained with DAPI (shown in blue). Multi-cell images were magnified 2.5x using Imaris software while single cell images were magnified 6X. The multi and single cell images were from separate experiments. Histograms represent the total fluorescence in whole cells and in the nuclei from at least 10 cells. Nuclear and cytosol region masks were created using Imaris software to determine the total fluorescent intensity corresponding to SPHK1 or SPHK2 in Z-stacks. Of note, quantifications of SPHK expression in this figure are "total intensity/cell" while in Figure 2A, SPHK expression is normalized by total protein, which does not equate to cell number since neoplastic cells have higher protein content.

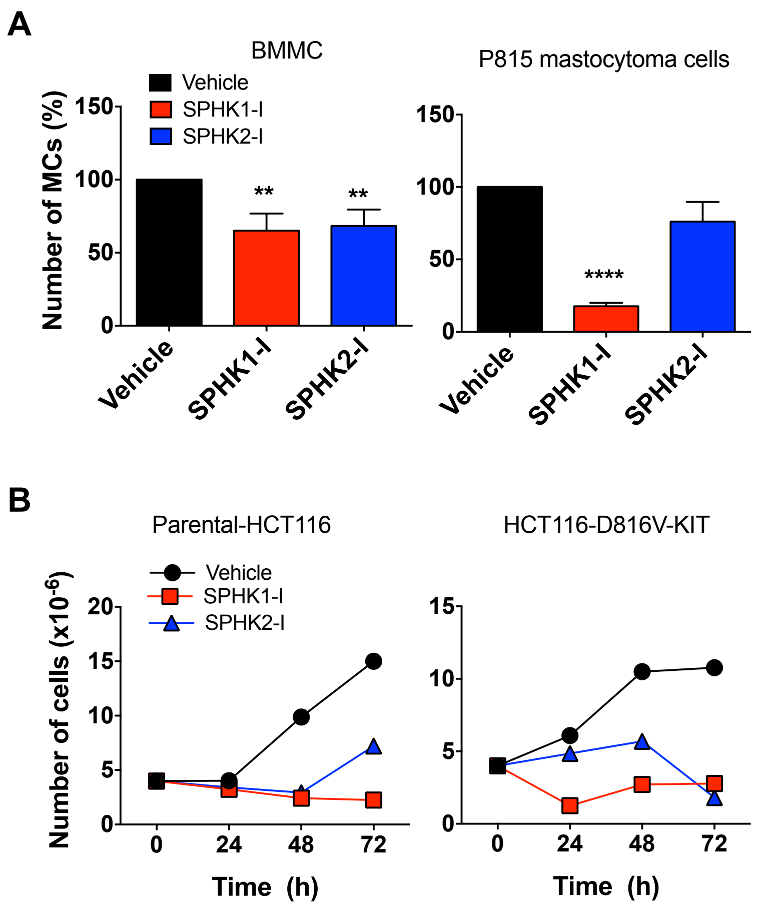

Figure S2- Effect of SPHK1 or SPHK2 inhibition on the growth of cells with D816V-KIT. (A) Reduction in the number of viable WT BMMC (A, left panel) or P815 murine mastocytoma cells with D816V-KIT (A, right panel) after treatment with SPHK1-I (5 μM) or SPHK2-I (50μM). Cells (0.5x10^5/well) were plated in 96 well plates, treated with vehicle or the inhibitors. Proliferation of cultures was measured after 3 days using Cyquant assay. The values in untreated controls at the end of the experiment was considered as 100% and the values in cells treated with the indicated inhibitors represent percentages compared to control values. Data are represented as mean±SEM of 3 independent experiments. (B) Effect of SPHK1-I or SPHK2-I in the growth of HCT116 colon cancer cells (left panel) or HCT116 where a mutation in KIT (D816V) was introduced (right panel). Viable cells were counted every day for 3 days. Data are from a single experiment out of three, and numbers are average of a duplicate determination.

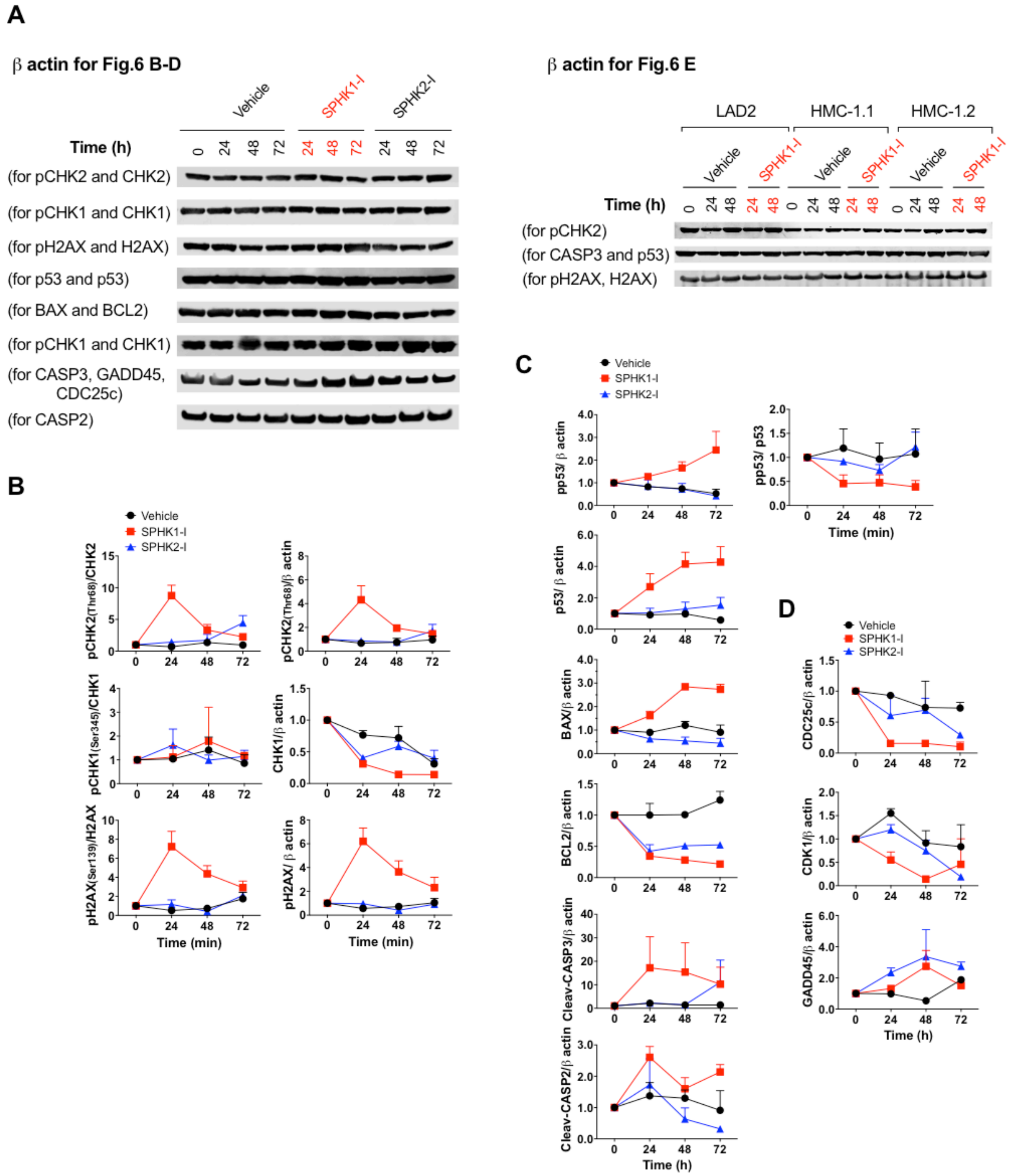

Figure S3- Quantification of the Western blots shown in Figure 6 B-D. (A) β-actin loading controls for the indicated Western blots shown in Figure 6 B-E. Full gels for the data shown in Figure 6E are shown in Supplemental Figure 6 (B-D) The band intensities in the blots shown in Figure 6B (B), 6C (C) and 6D (D) was determined using an Odyssey Image Studio software. The near-infrared fluorescent signal of the specific bands was normalized by the intensity of bands in the loading control as indicated. Data are the average values ±SEM of 3 independent experiments.

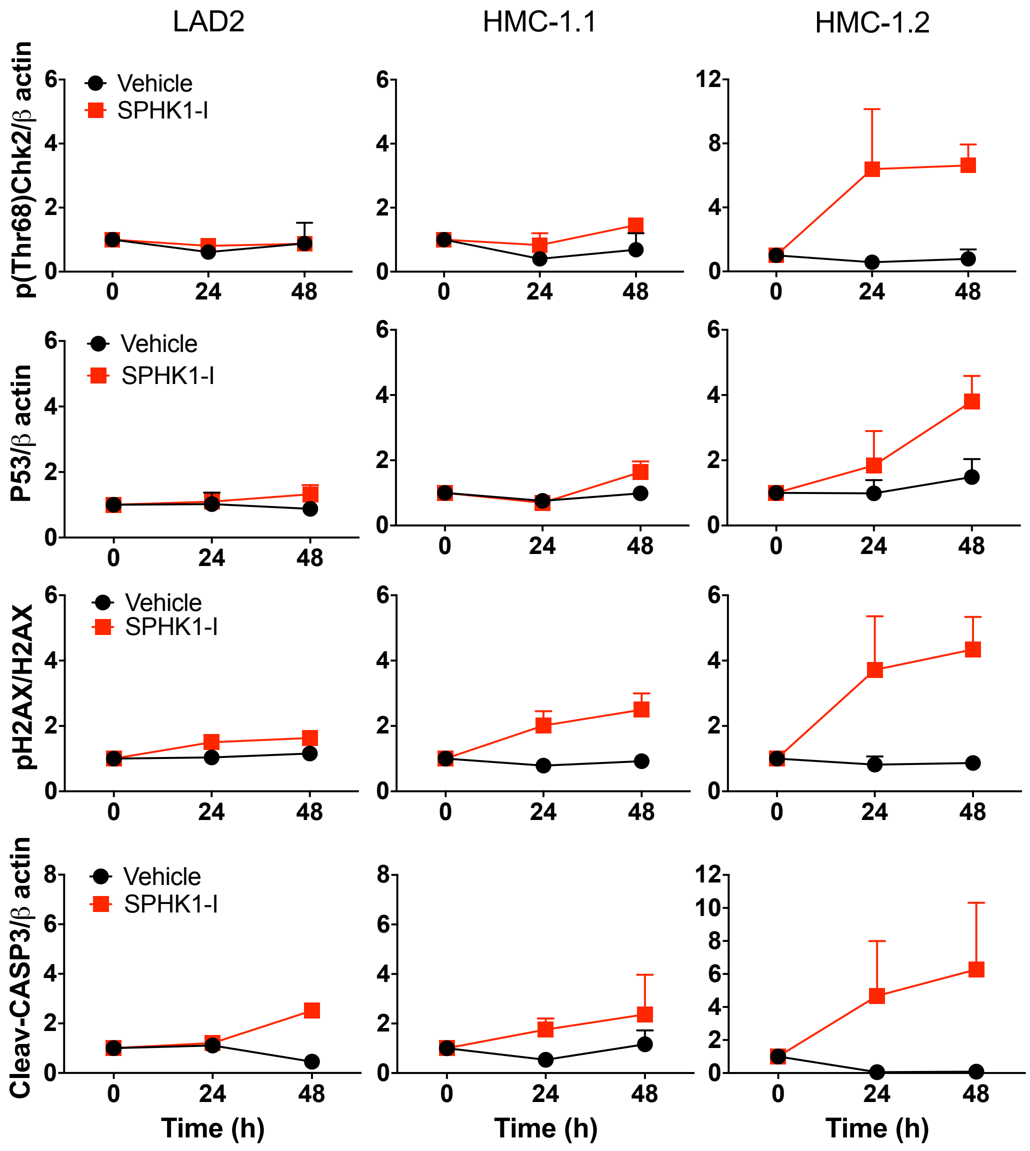

Figure S4- Quantification of the Western blots shown in Figure 6E. The band intensity in the blots shown in Figure 6E was determined using an Odyssey Image Studio software. The near-infrared fluorescent signal of the specific bands was normalized by the intensity of bands in the loading control as indicated. Data are the average values ±SEM of 3 independent experiments.

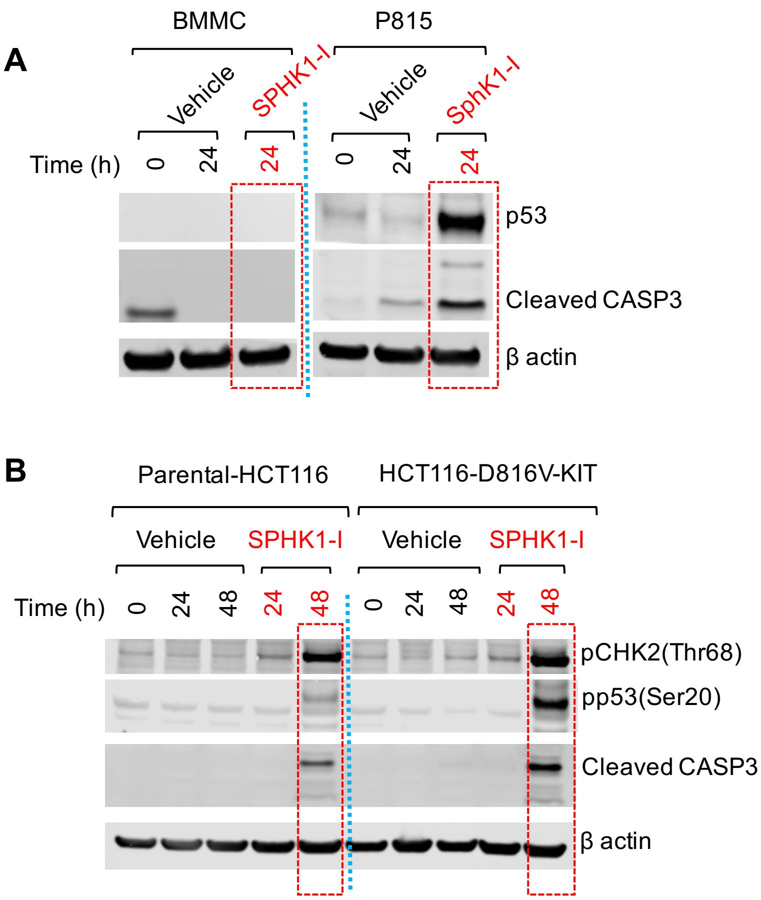

Figure S5- Effects of SPHK1-I on the activation of the DDR in cells with D816V-KIT. (A) Western blots analysis of lysates from BMMC or P815 murine mastocytoma cells with D816V-KIT, as indicated, after treatment with SPHK1-I (10 µM) or vehicle for 24 h. Note that the anti-human phospho-antibodies for CHK2 and phospho(Ser 20)-p53 do not recognize the respective mouse phosphoproteins and no anti mouse phospho-antibodies were available, so those are not shown. (B) Western blots analysis of lysates from HCT116 cells or HCT116 with D816V-KIT after treatment with SPHK1-I (10 µM) or vehicle for 24 or 48 h, as indicated. Blots show changes in the activation/levels of key effectors in the DDR shown in HMC-1.2. All blots are from a representative experiment of at least three separate experiments. Each membrane was probed with β-actin to demonstrate equal loading. Treatment of P815, as in HMC1.2, with SPHK2-I (50 µM) did not cause any changes in p53 (not shown) and thus this inhibitor was not further tested.

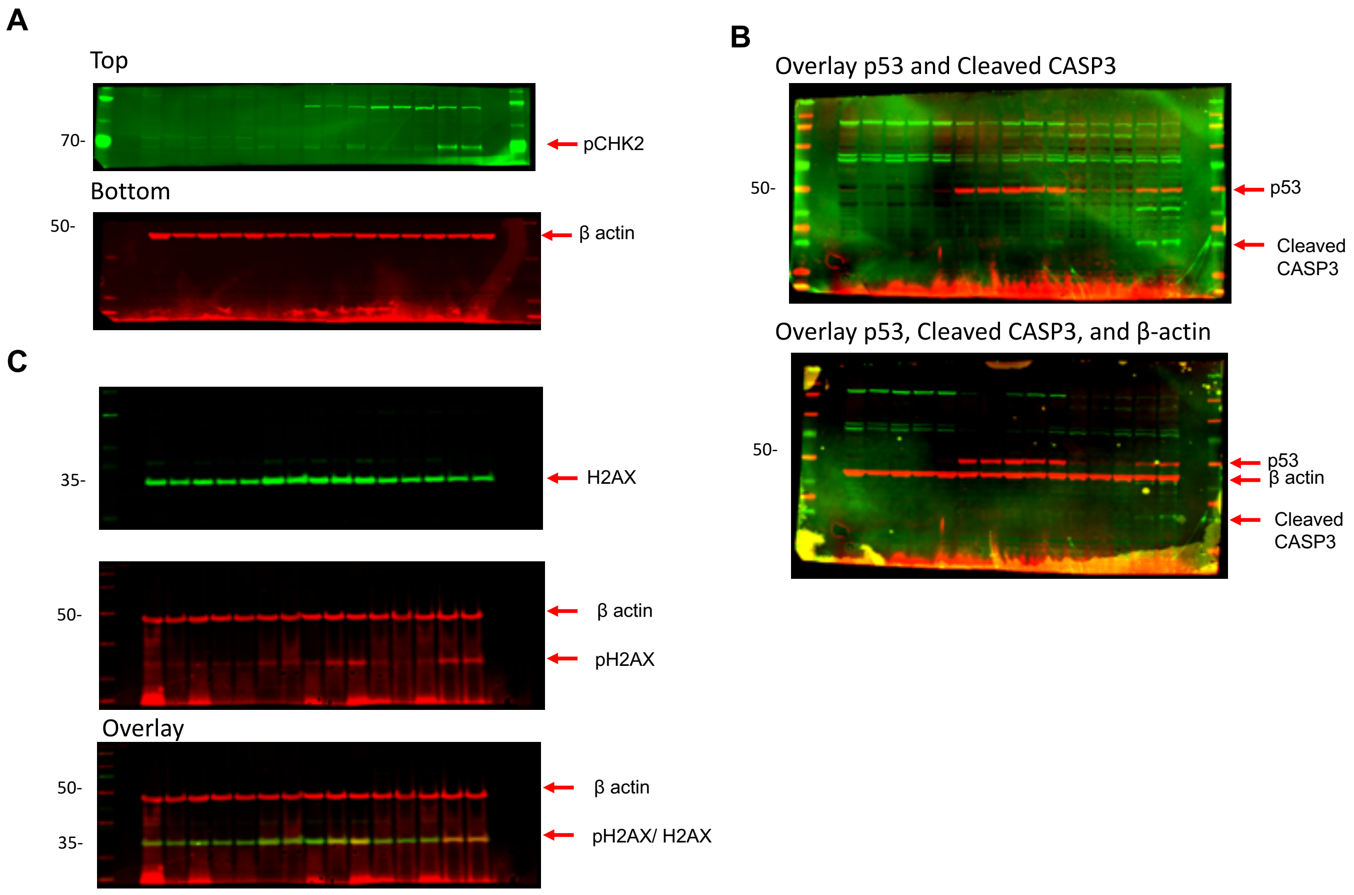

Figure S6- Raw Infrared images of the western blots shown in Figure 6E. (A) The Western blot membrane was cut into strips. The top of the membrane shows the bands for pCHK2(Thr68) in green and the bottom, the corresponding β-actin in red. (B) Whole membrane showing the bands for Cleaved CASP3 in green, and p53 and β-actin in red. (C) Bands for total H2AX in green and pH2AX and β-actin in red. An overlay of red and green infrared images is also shown. In B and C, membranes were probed with the corresponding antibodies sequentially, being anti-β-actin the last antibody used. Images were obtained with an Odyssey imaging system (Licor) and bands were visualized using anti-rabbit IgG 800CW (green) or anti-mouse IgG 680 RD (red) secondary antibodies.
