# Supplementary material for: Targeting Sphingosine Kinase Isoforms Effectively Reduces Growth and Survival of Neoplastic Mast Cells With D816V-KIT
Source: Front Immunol. 2018 Mar 28;9:631. doi: 10.3389/fimmu.2018.00631 (PMC5883065; doi:10.3389/fimmu.2018.00631)
Supplement: Supplementary file 2 [file table_1.PDF]

Supplementary Table I- Characteristics of the study subjects with systemic mastocytosis

	**WHO Classification**	**Age**	**Gender**	**Serum Tryptase (ng/ml)**	**Bone Marrow MC aggregates**	**% MC in Bone Marrow**	**D816V frequency in BM/PB**	**UP**	**Organomegaly**
Pat.1	ISM, eosinophilia	41	Male	291.0	Y Multiple	25-30%	13.44%/14.3 %	Y	N
Pat.2	SSM	54	Female	105.0	Y Multiple	25%	0.32%/0.27 %	Y	Y Hepatomegaly
Pat.3	ISM, eosinophilia	70	Female	334.0	Y Large	25%	31.08%/37.34%	Y	N
Pat.4	SSM	53	Female	479.0	Y Fibrosis, round MC	50-60%	1.413%/0.391%	N	Y Splenomegaly

Abbreviations: WHO, World Health Organization; Pat, patient; BM, bone marrow; PB, peripheral blood; ISM, indolent systemic mastocytosis; SSM, smoldering systemic mastocytosis. Normal tryptase level is < 11.5 ng/ml.
