# Supplementary material for: Targeting Sphingosine Kinase Isoforms Effectively Reduces Growth and Survival of Neoplastic Mast Cells With D816V-KIT
Source: Front Immunol. 2018 Mar 28;9:631. doi: 10.3389/fimmu.2018.00631 (PMC5883065; doi:10.3389/fimmu.2018.00631)
Supplement: Supplementary file 3 [file table_2.PDF]

**Supplementary Table II-** List of top canonical pathways affected by suppression of SPHK1 or SPHK2 activities in HMC-1.2 cells.

| Top Canonical Pathways | SphK Inhibitor | Z-score | P-value | Ratio | Molecules in the pathway |
|---|---|---|---|---|---|
| Cyclins and Cell Cycle Regulation | SPHK1-I | ↓ (-1.9) | $5x10^{-15}$ | 0.103 | CCNE1,CDKN1A,CDK6,CDK4, CDK1,CDK2,SKP2,CDC25A |
| | SPHK2-I | ↓ (-3.2) | $2x10^{-21}$ | 0.154 | TP53,CCNA2,CCNE1,TFDP1,ABL1,CCNB2,CDKN1B,CDK1,CDK2,SKP2,CDC25A,CCNB1 |
| Estrogen-Mediated-S-phase Entry | SPHK1-I | ↓(-2.7) | $1x10^{-16}$ | 0.292 | CCNE1,CDKN1A,CDK4,CDK1, CDK2,SKP2,CDC25A |
| | SPHK2-I | ↓(-2.7) | $2x10^{-17}$ | 0.333 | CCNA2,CCNE1,TFDP1,CDKN1B,CDK1,CDK2,SKP2,CDC25A |
| G1/S Checkpoint Regulation | SPHK1-I | ↑ (1.6) | $2x10^{-13}$ | 0.109 | CCNE1,CDKN1A,CDK6,CDK4, CDK2,SKP2,CDC25A |
| | SPHK2-I | ↑ (2.5) | $1x10^{-13}$ | 0.125 | TP53,CCNE1,TFDP1,ABL1,CDKN1B,CDK2,SKP2,CDC25A |
| **ATM Signaling** | SPHK1-I | (0) | $3x10^{-17}$ | 0.112 | RAD51,GADD45A,RAD9A,CDKN1A,CDK1,CHEK2,CDK2,CDC25A,CHEK1 |
| | SPHK2-I | ↑ (2.1) | $6x10^{-15}$ | 0.112 | TP53,GADD45A,ABL1,CCNB2, CDK1,CDK2,CDC25A,CHEK1, CCNB1 |
| **Role of CHK Proteins in Cell Cycle Checkpoint Control** | SPHK1-I | ↓ (-.8) | $6x10^{-14}$ | 0.127 | RAD9A,CDKN1A,CDK1,CHEK2,CDK2,CDC25A,CHEK1 |
| | SPHK2-I | (0) | $3x10^{-8}$ | 0.09 | TP53,CDK1,CDK2,CDC25A,CHEK1 |

Fold changes in gene expression shown in Table II were used for analysis using Ingenuity Pathway Analysis (IPA) using a cut-off for up-regulated and down-regulated gene transcripts of 1.5 fold ($p<0.05$).
